# Trabeculectomy or modified deep sclerectomy in juvenile uveitic glaucoma

**DOI:** 10.1007/s12348-011-0039-5

**Published:** 2011-09-08

**Authors:** Carsten Heinz, Jörg M. Koch, Arnd Heiligenhaus

**Affiliations:** 1 Department of Ophthalmology, St. Franziskus-Hospital Muenster, Hohenzollernring 74, 48145 Muenster, Germany; 2 University Duisburg Essen, Hufelandstr. 55, 45122 Essen, Germany

**Keywords:** Uveitis, Glaucoma, Child, Trabeculectomy, Sclerectomy

## Abstract

**Purpose:**

The purpose of this study is to report the effectiveness of trabeculectomies (TE) and modified deep sclerectomies (mdS) in a group of patients with juvenile uveitic secondary glaucoma.

**Methods:**

This is a retrospective analysis of 16 TE and eight mdS.

**Results:**

Postoperatively, an IOP reduction to 11.6 ± 4.7 mmHg was achieved in the TE group and to 18.5 ± 11.4 mmHg in the mdS group (*p* = 0.045). In the TE group, 14 patients showed postoperative success, one limited success and another was a failure compared to four successes and four failures in the mdS group (*p* = 0.041). The mean number of complications was 1.25 ± 1.49 in the TE group and 0.38 ± 0.74 after mdS (*p* = 0.11). In the mdS group, four patients (50%) needed additional glaucoma surgery compared to one TE patient (*p* = 0.023).

**Conclusion:**

Both surgical techniques showed a marked reduction of IOP. Trabeculectomy has a higher probability of achieving success and lowering IOP.

## Introduction

Management of secondary uveitic glaucoma in children can be quite challenging. The reported incidence of glaucoma in childhood uveitis varies profoundly in the literature, with figures up to 35% [1–4]. Little data are available on the success rates of topical therapy alone. In two studies on eyes predominately with JIA-associated anterior uveitis, 17% and 26% of patients had controlled intraocular pressure (IOP) with topical therapy alone [5, 6]. The rate of surgery was also comparable with 63% and 59% in these studies [5, 6]. No widely accepted recommended surgical approach exists so far to treat this group of secondary juvenile glaucoma patients. The aim of this study is to compare success rates of two glaucoma filtering surgery procedures, especially to examine the value of the non-penetrating approach, in a group of juvenile uveitis patients with secondary glaucoma. Secondary outcome measure was the number of additional glaucoma surgery.

## Patients and methods

All consecutive trabeculectomies (TE) or modified deep sclerectomies (mdS) performed from 2001 to the end of 2009 in patients with uveitis with onset before the age of 16 years and secondary glaucoma were retrospectively analysed. All patients underwent the surgical procedure after topical and systemic antiglaucomatous medication failed to prevent sustained IOP above 24 mmHg or following the detection of typical glaucomatous optic disc morphology by funduscopy. The study design complies with the Declaration of Helsinki ethical standards. Our institutions do not need approval of the local ethics committees for chart review studies. Uveitis was classified according to the recommendations of the International Uveitis Study Group and is also in accordance with the recent modifications [7, 8].

Demographic data, anatomic classification, association with uveitis, best corrected visual acuity, onset of uveitis and the time at which glaucoma was diagnosed were recorded for each patient. The presence of typical uveitis-related complications were documented, e.g. band keratopathy, cataract formation, secondary glaucoma (presence of typical glaucomatous disc cupping as measured by biomicroscopy), posterior synechies, vitreous opacities, macular oedema, ocular hypotony, phthisis or retinal detachment. In addition, the number of topical and systemic antiglaucomatous and immunosuppressive medications administered was documented. In addition, the number of previous surgeries, especially transscleral diode cyclophotocoagulation (TDCPC), and lens status were evaluated. Assessment of IOP was done by Goldman applanation tonometry after topical anaesthesia between 8 and 12 a.m. Patients were also on their regular scheme for application of IOP-reducing topical medications. Surgery was performed in all patients after quiescence of inflammation was achieved for at least 2 months. All patients received topical unpreserved dexamethasone phosphate 1% eye drops five times daily for 1 week prior to surgery to reduce conjunctival inflammation [9]. Topical and systemic antiglaucomatous medications had to be continued until surgery due to the high range of IOP. In children with bilateral secondary glaucoma, only the first operated eye was included.

One surgeon performed all surgical procedures. In all of these patients, the conjunctival area for bleb formation had been omitted from TDCPC. Trabeculectomy was performed in a standard fashion, with a fornix-based conjunctival and limbus-based scleral flap (3 × 3 mm). Mitomycin C (0.2 mg/ml) was applied to the bare sclera for 1 min before preparation of the scleral flap. Trephination was performed with a 1.5-mm trephine, followed by basal iridectomy. The sclera flap was adjusted with four non-absorbable 10-0 nylon stitches, and wound closure was performed in two layers with 10-0 polyglyctan sutures.

In modified sclerectomy, the conjunctiva was opened fornix based with a 6-mm incision. Mitomycin C (0.2 mg/ml) was applied on the bare sclera for 1 min before preparation of the scleral flap. A limbus-based scleral flap was prepared. Following this, a deep scleral lamella with de-roofing of Schlemm's channel was performed. In order to avoid vitreous prolapse, two circumscribed punctures were placed from the Schlemm's channel into the anterior chamber, lateral to the sclerectomy. The scleral flap was adjusted with non-absorbable 10-0 nylon stitches. The conjunctiva was closed with 10-0 polyglyctan sutures.

Following surgery, both groups were treated with atropine 1%; prednisolone acetate 1% and gentamicin eye drops. Topical and anti-inflammatory medication was continued after surgery, and dosages were adapted to the course of inflammation.

An IOP of 21 or lower without glaucoma medication was defined as a success. An IOP of 21 or lower with a requirement for antiglaucomatous medication was defined as a limited success; all other IOPs were defined as failures. An IOP of 6 mmHg and lower was also defined as failure. A second IOP limit for definition of success was set at 15 mmHg as recommended by the World Glaucoma Association [10]. Minimum follow-up time after surgery was 12 months.

The Fisher's exact or chi-squared test was used for categorical values of complications and the Student's *t* test for linear values. Hazard ratio and logrank test were calculated for the survival analysis. A significance level of 5% was used for all studies.

## Results

A total of 16 TE and eight mdS were performed in children (19 girls) with uveitis onset before the age of 16. Age at time of surgery was 12.7 ± 3.8 years, and follow-up took place 25.1 ± 13.2 months (range 12–74 months) after surgery in the whole study population. The mean association with juvenile idiopathic arthritis as an underlying disease was 79% within the whole group. There were no differences between the groups concerning the kind of topical antiglaucomatous or immunosuppressive therapy before surgery (Table 1). None of the patients had previous filtering surgery or implantation of glaucoma drainage devices in the past. Detailed information on medical and surgical glaucoma treatment before study procedures is also included in Table 1.

**Table 1:** Patient's demographics

Demographics	TE	mdS	*p* value
Number of patients	16	8	
Gender			
Female/male	14/2	5/3	0.37
Age in years			
At time of surgery	13.6	11.9	0.8
At uveitis diagnosis	5.0	4.7	0.11
Follow-up (months)	21.5	29.4	0.21
Uveitis association			
JIA	12	7	
Herpetic keratouveitis	0	1	
Idiopathic	4	0	0.13
Anatomic localization			
Anterior	12	7	
Intermediate	4	0	
Panuveitis	0	1	0.13
Lens status			
Phakic	11	1	
Pseudophakic	2	2	
Aphakic	3	5	0.03
Current therapy			
Topical glaucoma therapy (number of drugs)	3.31	3.38	0.8
Systemic immunosuppression	14	6	0.57
TDCPC	14	6	0.52
Systemic CAI	10	6	1

*JIA* juvenile idiopathic arthritis, *TDCPC* transscleral diode cyclophotocoagulation, *CAI* carbonic anhydrase inhibitors, *TE* trabeculectomy, *mdS* modified deep sclerectomy

**Fig. 1:**
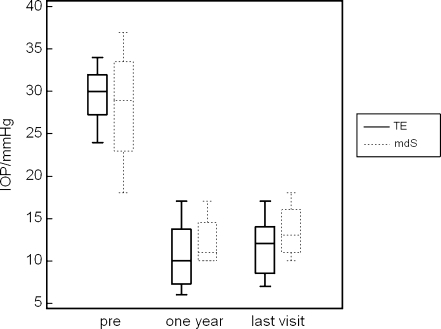
Intraocular pressure before surgery (*pre*), 1 year after surgery and at the last visit of patients without failure (limit ≤21 mmHg) at the last visit. *TE* trabeculectomy (*n* = 15), *mdS* modified deep sclerectomy (*n* = 4)

IOP before filtering surgery was similar in both groups, with 28.3 ± 5.7 mmHg in the TE group and 30.3 ± 6.3 mmHg in the mdS group (*p* = 0.44). One year postoperatively or at the date of failure, an IOP reduction to 11.6 ± 4.7 mmHg was achieved in the TE group and to 18.5 ± 11.4 mmHg in the mdS group (*p* = 0.045). Figure 1 shows the IOP values before surgery, 1 year after surgery and at the last visit of patients without failure (≤21 mmHg or below with or without medication) at the last visit. The mean IOP for patients with a success or limited success 1 year after surgery and at the last visit were slightly higher in the mdS group without significance (*p* = 0.42 and at last visit *p* = 0.36)

Success (limit IOP ≤21 mmHg) was achieved in 14 (88%) patients after TE as compared to 4 (50%) patients in the mdS group at the last visit. Surgical failure was observed in one patient (6%) from the TE group, and in another four (50%) cases in the mdS group (*p* = 0.041). Success rate 1 year after surgery was comparable with identical data in the TE group and five successes and three failures in the mdS group (*p* = 0.13) Using the more rigid classification at the last visit with IOP ≤15 mmHg as limit, 12 (75%) TE patients achieved a success compared to 3 (38%) in the mdS group. Limited success was found in one (6%) patient in the TE group, while three (19%) patients in the TE group and five (63%) in the mdS group failed (*p* = 0.092). None of the patients failed due to hypotony (IOP ≤6 mmHg). In those patients with success, the typical thin avascular blebs could be observed. Subgroup analysis of success rate (IOP ≤21 mmHg) and lens status revealed are presented in Table 2. All failures were JIA children and were associated with profound subconjunctival scarring after glaucoma surgery.

**Table 2:** Success rates after surgery, subgroups after lens status Pearson and Fisher test, success IOP ≤21 mmHg without antiglaucomatous therapy, limited success IOP ≤21 mmHg with antiglaucomatous therapy and failure all others

Number		TE (16)	mdS (8)	*p* value
All	Success	14	4	
	Limited success	1	0	
	Failure	1	4	0.041
Phakic	Success	10	1	
	Limited success	1	0	1.0
Aphakic	Success	2	3	
	Failure	1	2	1.0
Pseudophakic	Success	2	0	
	Failure	0	2	0.33

*TE* trabeculectomy, *mdS* modified deep sclerectomy

**Fig. 2:**
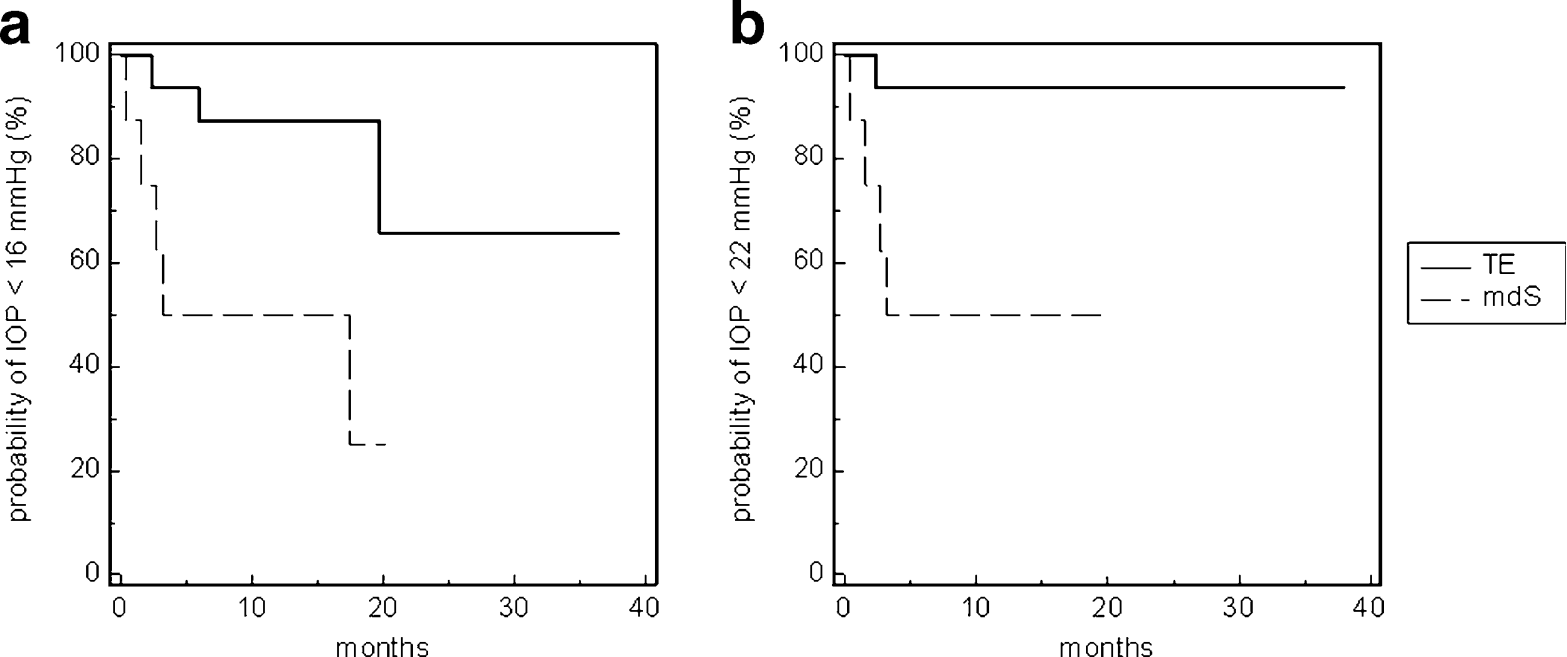
Kaplan–Meier survival curve for IOP ≤21 mmHg (**a**) and for IOP ≤15 mmHg (**b**) in the patients after trabeculectomy (*TE*) and modified deep sclerectomy (*mdS*)

Figure 2 shows the survival rate for both IOP classifications. Using the classification of an IOP of 15 mmHg or below, four out of five failures occur in the mdS group within the first year, while failures in the TE group are distributed over the complete follow-up period. The hazard ratio (HR) for the limit of 15 mmHg was 0.16 with a 95% confidence interval (CI) of 0.033 to 0.766 (*p* = 0.02 logrank test). For the 21-mmHg limit, the HR was 0.08 with CI of 0.012 to 0.573 (*p* = 0.01).

Visual acuity did not differ significantly between the two groups before (LogMAR TE group 0.3 ± 0.3 vs. mdS group 0.43 ± 0.32; *p* = 0.32) and after surgery (LogMAR TE group 0.38 ± 0.35 vs. mdS group 0.6 ± 0.32; *p* = 0.14). Also the number of uveitis relapses within the first year after surgery did not differ between the two groups with six flares in the TE and two flares in the mdS group (*p* = 0.66). The mean number of complications, including a prominent Tenon cyst, a shallow anterior chamber, choroidal detachment combined with hypotony, vitreous prolapse into the trephination and papilloedema, was 1.25 ± 1.49 in the TE group and 0.38 ± 0.74 in the mdS group (*p* = 0.11). A shallow anterior chamber and a choroidal detachment were the most frequent complications in the TE group (Table 3). Additional surgery after initial TE or mdS was dived into limited surgical interventions to achieve controlled IOP or in additional glaucoma procedures. In the TE group, limited surgical interventions were necessary in eight (50%) patients compared to none in the mdS group (*p* = 0.022). One of those needed another glaucoma procedure to achieve IOP below 21 mmHg later on. In the mdS group, the need for additional glaucoma surgery was 50% (four patients, *p* = 0.023; Table 4).

**Table 3:** Number and type of complications in both groups

Complications	TE	mdS
Tenon cyst	1	1
Vitreous prolapse	2^a^	
Shallow anterior chamber	7	1
Choroidal detachment	8	1
Papilloedema	4	
Cataract formation^b^	5	1

*TE* trabeculectomy, *mdS* modified deep sclerectomy

^a^Both were aphakic

^b^No proven association with surgical intervention

**Table 4:** Numbers and type of surgical interventions for pressure control or additional glaucoma surgery

	TE	mdS	*p* value
Number of patients with no procedure	8	4	
Number of patients with limited surgical interventions to achieve controlled IOP	8	0	
Downsizing of filtering bleb	4		0.022
Suture lysis	1		
Needling of Tenon cyst	1		
Removal of vitreous strands	2		
Number of patients with additional glaucoma operation	1	4	
Second trabeculectomy	1	1	0.023
TDCPC		1	
Molteno implant		1	
Goniosynechiolysis		1	

In one TE patient needling and re-trabeculectomy were performed

*TDCPC* transscleral diode cyclophotocoagulations, *TE* trabeculectomy, *mdS* modified deep sclerectomy

## Discussion

At present, there is no generally accepted or recommended universal surgical approach for the management of juvenile uveitic secondary glaucoma. Surgical treatment modalities include trabeculectomy with MMC, goniotomy, trabeculodialysis implantation of drainage devices and cyclodestructive procedures. The success rates reported for these techniques at the end of profoundly diverse follow-up times vary between 60% and 90% [5, 11–14]. One study focused on JRA patients, which is a high risk glaucoma group comparable to our patients. In this group, which is known for its high failure rate after surgery, conventional filtering surgery was able to control IOP in 57%, while trabeculectomy with MMC controlled IOP in the remaining four cases [5]. Cyclodestructive procedures, such as transscleral diode laser cyclophotocoagulation, proved ineffective as a primary surgical approach in JIA-associated uveitis and secondary glaucoma as the success rate was only 32% after 1 year [15].

The value of non-penetrating glaucoma surgery for the management of uveitic glaucoma has only been described in few reports on adult patients. Overall success rates (complete and qualified success) range from 87% to 100%. A favourable aspect of this technique is that complications (in these cases, lens opacities, reversible hypotony, hyphema and bleb encapsulation) occur infrequently [16–18]. We used a slightly modified standard deep sclerectomy to obtain a conventional transscleral deep filtration and combined it with circumscribed goniotomies to increase the filtration. In order to minimize the risk of vitreous prolapse under the flap in aphakic eyes, the incisions on the inner wall of Schlemm's canal were placed at each side. We speculated that this might prevent the need for subsequent goniopuncture, a procedure commonly required after deep sclerectomy [19]. Until now, no studies have been available presenting data on non-penetrating glaucoma surgery and standard trabeculectomy in uveitis patients, especially in children. Souissi and co-workers have published their data on deep sclerectomy and on trabeculectomy in two separate papers in adults. Their groups consisted of 8 patients in the paper on deep sclerectomy, and another 17 in the trabeculectomy paper. Patients were aged 56.9 and 48.1 years, respectively. In the deep sclerectomy group, the procedure was successful in 88% of the patients, compared to 65% in the trabeculectomy group with a follow-up of 42 and 52 months [18, 20].

As reported by others, aphakic children had a higher failure rate after glaucoma surgery. In a group of patients with different entities of juvenile glaucoma, the failure rate after trabeculectomy with MMC in aphakic children was as high as 60% [21]. Another study analysed the outcome after goniotomies in chronic childhood uveitis. The success rate was only 36% (4 eyes) in aphakic patients, while 86% (25 eyes) of the procedures were successful in the phakic group [11]. In our retrospective setting, there has been a bias towards a certain surgical technique, which also results in uneven balance of our two groups. The aphakic children in this series were treated more frequently with the modified deep sclerectomy in order to avoid vitreous in the trephination. We are aware that comparison of the two reported groups is therefore limited in some aspects but still allows comparison of different aspects of these two surgical procedures.

One prospective randomized trial exists that compares these two different surgical techniques in adults with primary open angle or pseudoexfoliation glaucoma [22]. In this study, both techniques provided sufficient IOP reduction without any statistical significant difference. In another study by the same authors, trabeculectomy appeared to be more suitable for higher IOP levels and longer life expectancies than deep sclerectomies [23]. Our results regarding IOP reduction in juvenile uveitic glaucoma are in accordance with these previous observations, as trabeculectomy with MMC was more effective than modified deep sclerectomy with MMC.

The overall rate of additional surgery including limited postoperative interventions and additional glaucoma surgery was similar in both groups. In the mdS group, four additional glaucoma procedures had to be performed compared to one in the TE group, while in TE group, eight patients needed limited surgical interventions to adjust IOP to levels between 10 and 20, including downsizing of the filtering bleb, suture lysis and needling procedure. Postoperative choroidal detachment and a shallow anterior chamber occurred in as many as 50% of the patients with TE. This hypotony is probably due to reduced aqueous production and increased uveoscleral drainage because of postoperative inflammation. It can be speculated that these complications might have accelerated the development of posterior synechies, cataract formation and macular abnormalities. Cataract surgery was necessary in 5 of 11 phakic children in the TE group during follow-up (mdS group: 0 of 1 phakic patient required this treatment), but it is impossible to say whether the cataract formation was induced through surgery or inflammation. The vitreous prolapse that occurred during the immediate postoperative period in two aphakic eyes was unfortunate. However, after surgical removal of the vitreous strands, the trephination hole was overt.

In summary, glaucoma surgery in children with secondary uveitic glaucoma still remains difficult. Our data suggest that IOP can be sufficiently reduced using standard trabeculectomy with MMC and deep sclerectomy with MMC, but TE with MMC may be more effective. However, additional surgeries to adjust IOP finally were common for both groups. For aphakic children, the modified sclerectomy described earlier appears to be a good technique for avoiding vitreous prolapse.
